# Supplementary material for: The C2A domain of synaptotagmin is an essential component of the calcium sensor for synaptic transmission
Source: PLoS One. 2020 Feb 7;15(2):e0228348. doi: 10.1371/journal.pone.0228348 (PMC7006929; doi:10.1371/journal.pone.0228348)

Raw Blot Images Figure 3 A,B

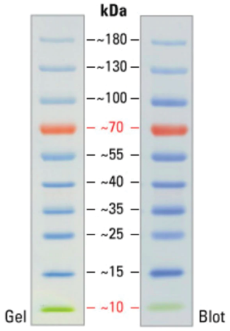

All blots were loaded left to right and imaged on an Epichemi3 Darkroom with Labworks Imaging Software. Ladder in all blots was Thermo Scientific PageRuler Prestained Protein Ladder. Lanes were only excluded (X) if the actin levels were 3 standard deviations from the mean actin level calculated within the blot. Genotypes: 1-*P[sytWT]*, 2- *P[sytA-ME]*, 3- *P[sytA-ME,FE]*

2016.06.30 A

70kDa

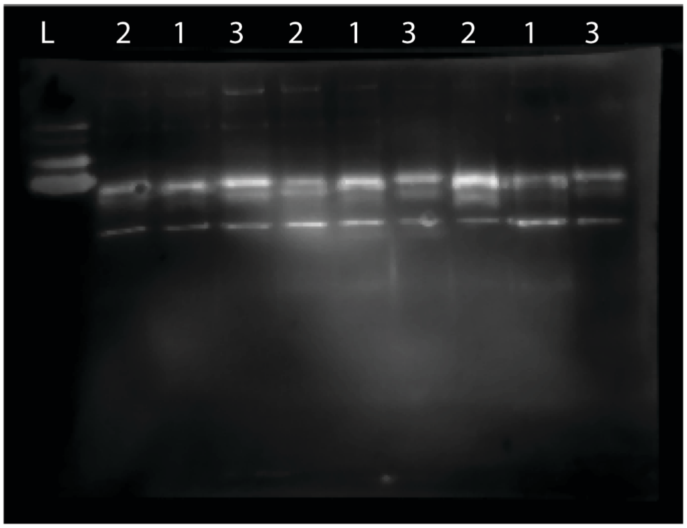

2016.07.08 B

70kDa

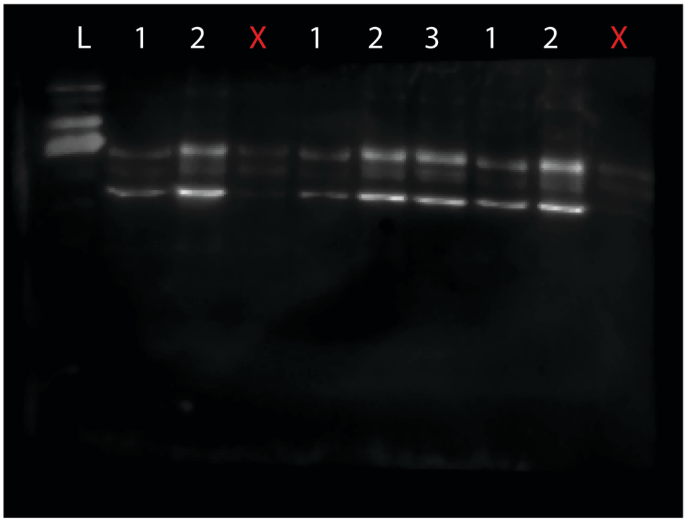

2016.07.14 A (Rep lanes for Fig 3A in red)

70kDa

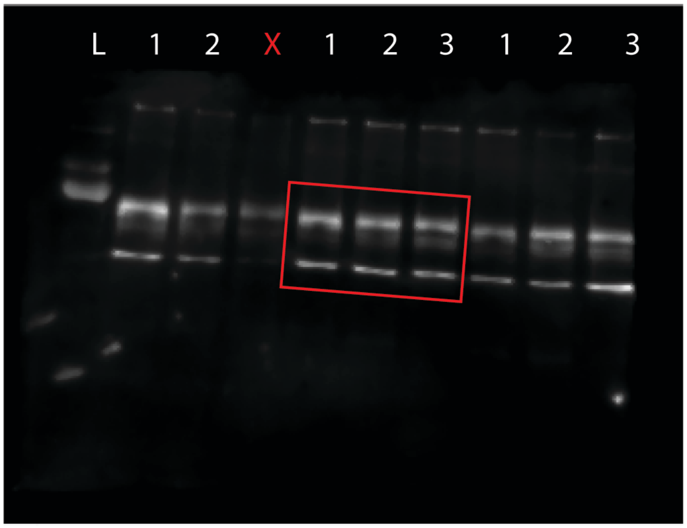

2016.07.14 B

70kDa

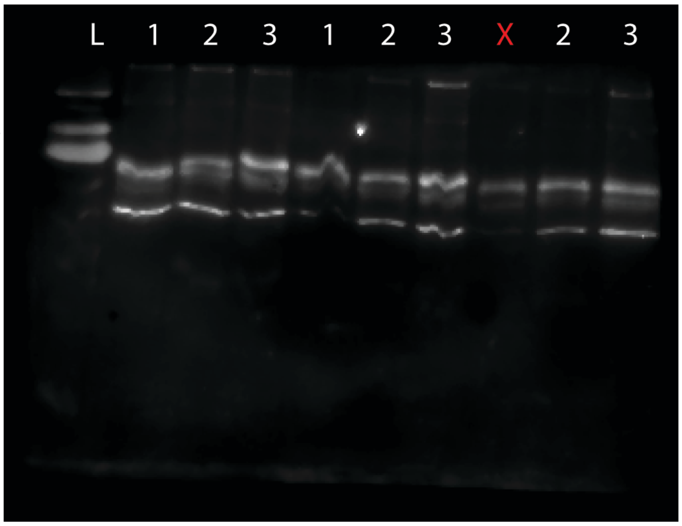

2016.07.20 A

70kDa

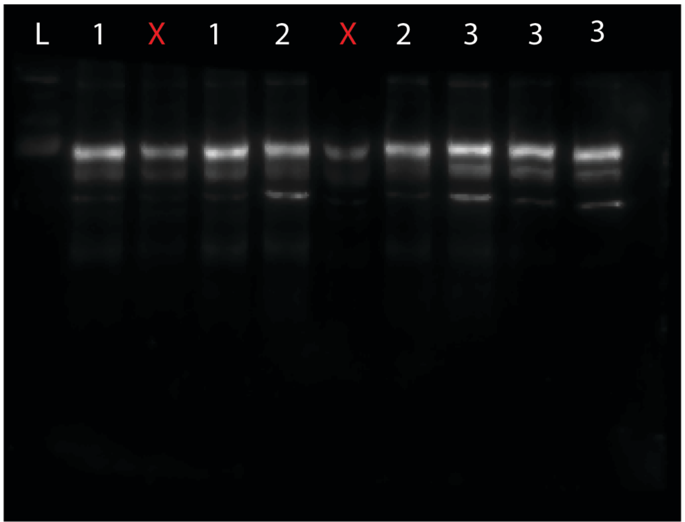

2016.07.20 B

70kDa

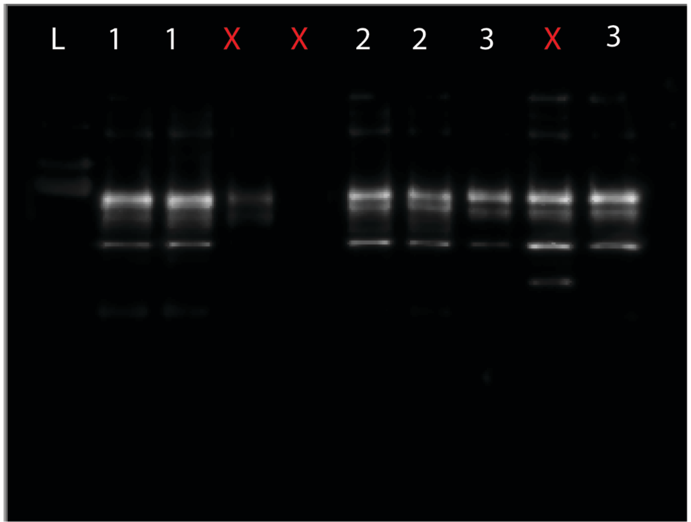

Supplement: S1 Raw images — (PDF) [file pone.0228348.s001.pdf]
