# Supplementary material for: The C2A domain of synaptotagmin is an essential component of the calcium sensor for synaptic transmission
Source: PLoS One. 2020 Feb 7;15(2):e0228348. doi: 10.1371/journal.pone.0228348 (PMC7006929; doi:10.1371/journal.pone.0228348)
Supplement: Attachment — Submitted filename: Bowers Reist Response to Reviewers.docx [file pone.0228348.s002.docx]

Thank you for the opportunity to resubmit a revised draft of our manuscript, “The C2A domain of synaptotagmin is an essential component of the calcium sensor for synaptic transmission.” We are grateful to the reviewers for their valuable feedback and that they “strongly support the publication of this work” and find it to be “timely and relevant.” We have addressed reviewer comments, as summarized below.

**Comments from Reviewer 1:**

*Major Comment 1:* “The author should include information on the calcium binding mutations in both C2A and C2B domain of synaptotagmin and discuss their result in context of those findings.”

*Response:* We have expanded our discussion of Ca^2+^ binding mutations in the C2A and C2B domains and the comparison to the hydrophobic mutations. (Lines 497-505)

*Major Comment 2:* “To the best of knowledge, disrupting calcium binding to C2A domain produced only partial effect as compared to near complete loss of function with comparable C2B mutation. How do the authors reconcile these findings? This should be discussed.”

*Response:* Thank you for pointing out that our discussion regarding the relative role of C2A vs. C2B was not clear enough. There is indeed a difference in neurotransmitter release depending on which domain has been mutated – C2B is still the predominant domain. We have expanded our discussion section to clarify this important point. (Lines 451-453, 507-517)

**Comments from Reviewer 2:**

*Major Comment 1:* “During the manuscript the authors refer only to “synaptotagmin”. It is mentioned at one point that it is synaptotagmin 1. Which other isoforms of syt are present in the neuromuscular junction and how could they potentially influence the results? Are the expression levels of other isoforms influenced by the mutations?”

*Response:* The reviewer brings up an important point that other synaptotagmin isoforms could impact synaptotagmin 1 mutations. In *Drosophila*, the only *syt* genes expressed at the neuromuscular junction are *syt 1* and *syt IV* and *syt IV* is concentrated in the postsynaptic cell. Unfortunately, the impact of *syt I* mutations on *syt IV* expression levels is unknown. We have expanded our discussion section to include this information. (Lines 574-585)

Again, we want to thank the reviewers for their thoughtful feedback, and we believe it has made the manuscript stronger.
